# Use of smart lacrimal probes

**DOI:** 10.4103/0301-4738.73702

**Published:** 2011

**Authors:** Mihir Kothari

**Affiliations:** Jyotirmay Eye Clinic and Pediatric Low Vision Center, Khopat, Thane, Maharashtra, India

Dear Editor,

Nasolacrimal duct probing is the treatment of choice for an unresolved congenital nasolacrimal duct obstruction (CNLDO).[1] We have found routine use of a nasal endoscope (5 mm, 0°) and *smart lacrimal probes* [Fig. 1A] as useful adjuncts to probing, especially for older children and repeat probings.[1,2]

**Figure 1:**
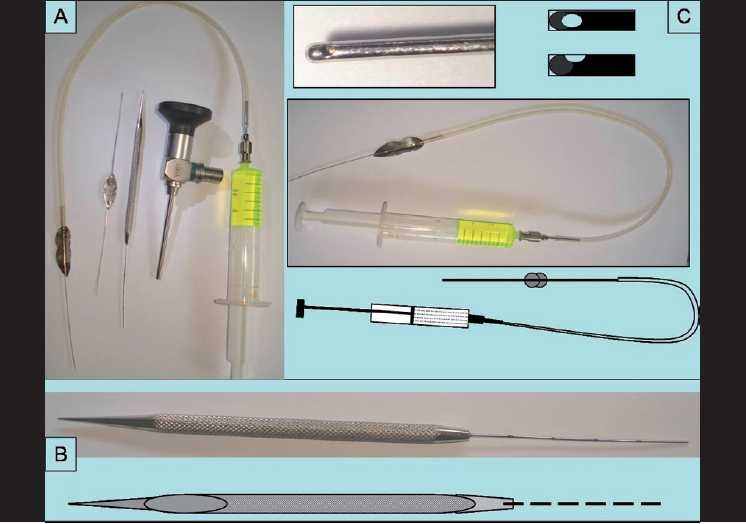
(A) Instruments used for performing the probing using nasal endoscope and the smart lacrimal probes; (B) graduated probe; (C) cannulated probe

However, there are two problems:

1. unavailability of endoscope at many eye hospitals and
2. difficulty in visualizing the probe in the nose.

I write this communication to bring to the notice of the readers, the utility of *smart lacrimal probes* that we have found valuable.

## 1. The graduated/measured probe [Fig. 1B]

Size of the lacrimal punctum in an infant is 0.3 mm.[3,4] However, even the thinnest probe measures 0.45 mm (Bowman 0000 lacrimal probe). To avoid injury, punctum should be dilated using one end of this instrument which is designed like a Nettleship’s punctum dilator.

On the other side, there is a 0.65-mm diameter stainless steel rod with markings at every 10 mm. After inserting the probe through the lacrimal punctum, the probe tip advances into the canaliculus. When the first mark at 10 mm approaches the punctum, the tip of the probe enters the lacrimal sac (canalicular length = 2 mm vertical + 8 mm horizontal).[5] This requires little manipulation to negotiate the probe through the common canaliculus.

After changing the direction of the probe, one continues to insert the probe. When the second marking (20 mm) approaches the lacrimal punctum, the tip of the probe approaches the bony canal. One can feel the resistance of the bony part of the nasolacrimal canal. With little manipulation, the probe tip enters the canal (the functional diameter of the bony canal is around 1 mm).[3,4,6] If the canal is stenotic, a gritty sensation is felt or the probe cannot be negotiated any further.

As the probe is advanced, third mark on the graduated probe (30 mm) approaches the punctum. One can feel slight resistance of the imperforate valve of Hasner and a sudden give away of the resistance as the probe tip enters the inferior meatus. When the probe is inserted beyond 30 mm mark, the tip of the probe can be visualized in the inferior meatus with the endoscope or can be felt as a metallic touch with another probe inserted in the inferior meatus.[2]

## 2. The cannulated/irrigating probe [Fig. 1C]

This is 0.7 mm in diameter with 0.3 mm cannulation. The end of the probe is knurled and tubular with a side opening for an easy entry and manipulation. The stainless steel cannulated rod is mounted on a holding member and connected to a silicon tube connected to a syringe containing fluorescein dye. Once the inferior meatus is reached, the fluid is injected which wells up in the inferior meatus momentarily and can be easily visualized by an endoscope or retrieved in suction catheter or on nasal gauze.

These probes do not improve the success rate by themselves but there are advantages. Graduated probe makes it easy to guess the location of the tip of the probe in the lacrimal system. When combined with the tactile feedback, good comprehension is achieved to decide what manipulation or maneuver may be required to proceed further and where exactly the stenosis or atresia may be located in the case of resistance or failure to proceed. It also helps to avoid unwarranted use of force when correct manipulation is required and it helps to decide when to stop probing further and look into the inferior meatus to confirm the presence of the probe tip there.

The cannulated probe is helpful to directly inject the fluorescein dye into the inferior meatus for an easy confirmation of the presence of the probe tip without having to insert a separate cannula. It also avoids regurgitation of the dye from the punctum and inadvertent injection of the dye in the pericanalicular or periorbital tissue.

At present, we have combined both the designs into one and used a graduated and cannulated probe. Every time I perform a lacrimal probing using this probe, my heart silently thanks Bangerter who first described the *hollow* probe,[7] Miyake who designed the graduated probes[4] and the Indian ophthalmic instrument manufacturer (Ankur metal works, Kolkota) who made them available at a very low cost.
